# A Genome-Wide Association Study To Understand the Effect of *Fusarium verticillioides* Infection on Seedlings of a Maize Diversity Panel

**DOI:** 10.1534/g3.119.400987

**Published:** 2020-03-10

**Authors:** Lorenzo Stagnati, Vahid Rahjoo, Luis F. Samayoa, James B. Holland, Virginia M. G. Borrelli, Matteo Busconi, Alessandra Lanubile, Adriano Marocco

**Affiliations:** * Dipartimento di Scienze delle Produzioni Vegetali Sostenibili, Università Cattolica del Sacro Cuore, via Emilia Parmense 84, 29122 Piacenza,Italy; † Seed and Plant Improvement Institute, Agricultural, Education and Extension Organization (AREEO), 51585 Karaj,Iran; ‡ Department of Crop & Soil Sciences, North Carolina State University, Raleigh, North Carolina 27695; § U.S. Department of Agriculture-Agricultural Research Service, Plant Science Research Unit, Raleigh, North Carolina 27695

**Keywords:** GWAS, SNPs, Artificial inoculation, *Fusarium verticillioides*, Maize

## Abstract

*Fusarium verticillioides*, which causes ear, kernel and stem rots, has been reported as the most prevalent species on maize worldwide. Kernel infection by *F. verticillioides* results in reduced seed yield and quality as well as fumonisin contamination, and may affect seedling traits like germination rate, entire plant seedling length and weight. Maize resistance to *Fusarium* is a quantitative and complex trait controlled by numerous genes with small effects. In the present work, a Genome Wide Association Study (GWAS) of traits related to *Fusarium* seedling rot was carried out in 230 lines of a maize association population using 226,446 SNP markers. Phenotypes were scored on artificially infected kernels applying the rolled towel assay screening method and three traits related to disease response were measured in inoculated and not-inoculated seedlings: plant seedling length (PL), plant seedling weight (PW) and germination rate (GERM). Overall, GWAS resulted in 42 SNPs significantly associated with the examined traits. Two and eleven SNPs were associated with PL in inoculated and not-inoculated samples, respectively. Additionally, six and one SNPs were associated with PW and GERM traits in not-inoculated kernels, and further nine and thirteen SNPs were associated to the same traits in inoculated kernels. Five genes containing the significant SNPs or physically closed to them were proposed for *Fusarium* resistance, and 18 out of 25 genes containing or adjacent to significant SNPs identified by GWAS in the current research co-localized within QTL regions previously reported for resistance to *Fusarium* seed rot, *Fusarium* ear rot and fumonisin accumulation. Furthermore, linkage disequilibrium analysis revealed an additional gene not directly observed by GWAS analysis. These findings could aid to better understand the complex interaction between maize and *F. verticillioides*.

Maize plants are attacked by several *Fusarium* species responsible for diseases such as root rot, stalk rot, seedling blight and ear rot, but *Fusarium verticillioides* (Sacc.) Nirenberg (synonyms *Fusarium moniliforme*, *Gibberella fujikuroi* MP-A or *Gibberella moniliformis*) (Bömke *et al.* 2008) has been reported as the most widespread fungal pathogen of maize worldwide (Leslie 1991; Danielsen *et al.* 1998; Chulze *et al.* 2000; Rahjoo *et al.* 2008). *Fusarium* infection can result in depleted seed yield and quality as well as fumonisin contamination. This fungus produces a wide range of mycotoxins that include fusaric acid, fusarins, and fumonisins (Desjardins and Proctor 2001). Fumonisins are the most prevalent (Munkvold 2003) and have been associated with esophageal cancer in humans, pulmonary edema in pigs, leukoencephalomalacia in horses and cancer-promoting activity in rats (Marasas 2001; Voss *et al.* 2002; Murillo-Williams and Munkvold 2008). Moreover, *F. verticillioides* can establish asymptomatically inside maize plants and kernels, impairing the possibility to easily detect affected grains (Munkvold and Desjardins 1997; Munkvold *et al.* 1997; Wilke *et al.* 2007).

*F. verticillioides* has available many infection pathways to reach plant tissues (Battilani *et al.* 2003), including the transmission from soil or the presence of seed borne *F. verticillioides* strains in all the plant tissues such as ears and kernels (Munkvold *et al.* 1997; Mesterházy *et al.* 2012). The frequency of fungal strain transmission from the seed depends on fungal characteristics, maize genotypes and environmental conditions (Wilke *et al.* 2007). Even though the complexity of maize-*F. verticillioides* interaction and the influence of seedling transmitted fungal strains on stalk, ear, and kernel rots as well as the fumonisin production was previously shown (Munkvold *et al.* 1997; Yates *et al.* 2003; Wilke *et al.* 2007; Williams *et al.* 2007), the potential genetic resistance to *Fusarium* seedling rot has not been widely examined yet.

Kernel infection by *F. verticillioides* can reduce seed germination of maize as well as vigor at different growth stages (Machado *et al.* 2013). The effect of *F. verticillioides* on the development of maize seedlings/plants under controlled conditions was previously evaluated (Machado *et al.* 2013). All traits measured (plant population stand, speed of seedling emergence, and height/weight of emerged plants) were negatively affected by the increasing fungal amounts present in the seeds (Machado *et al.* 2013). Recently, a genome-wide association study (GWAS) using artificially inoculated maize kernels allowed the identification of several SNPs and candidate genes significantly associated with *Fusarium* resistance at the seedling stage (Stagnati *et al.* 2019).

The cultivation of resistant germplasm is an efficient way to reduce yield loss and mycotoxin contamination, however, no maize genotypes immune to *Fusarium* infection have been identified and many commercial hybrids have less resistance than desired (Lanubile *et al.* 2010; Zila *et al.* 2013). Resistance to *F. verticillioides* in maize, which was mainly focused on *Fusarium* ear rot (FER), has been studied in different surveys based on artificial inoculation in field (Drepper and Renfro 1990; Reid and Zhu 2005; Reid *et al.*, 2009; Lanubile *et al.* 2011, 2017; Zila *et al.* 2013; Maschietto *et al.* 2017), greenhouse (Danielsen *et al.* 1998; Lanubile *et al.* 2013, 2014a; Maschietto *et al.* 2016) and laboratory trials (Ju *et al.* 2017; Atabaki *et al.* 2018). Moreover, in order to develop genetically resistant germplasm to *Fusarium* researchers carried out Quantitative Trait Locus (QTL) mapping analysis and GWAS by testing several distinct population structures (McMullen *et al.* 2009; Yang *et al.* 2010; Zila *et al.* 2013, 2014; Warburton *et al.* 2013; Chen *et al.* 2016; Ju *et al.* 2017; Maschietto *et al.* 2017; Gaikpa and Miedaner 2019; Samayoa *et al.* 2019; Septiani *et al.* 2019).

In the present study, the Rolled Towel Assay (RTA) screening method was applied to a maize association panel (Flint-Garcia *et al.* 2005) for evaluating the effect of *F. verticillioides* infection on the vegetative response of maize seedlings, considering plant seedling length (PL), plant seedling weight (PW) and germination rate (GERM). The panel was previously analyzed for resistance to *Fusarium* infection of seedlings (FIS; Stagnati *et al.* 2019). In order to identify SNPs putatively associated with PL, PW and GERM, with and without kernel infection by *F. verticillioides*, GWAS and linkage disequilibrium (LD) analysis were performed.

## Materials and Methods

### Maize germplasm, kernel inoculation and phenotyping

The maize core diversity panel, sometimes referred as the “Goodman” association panel (Flint-Garcia *et al.* 2005; Zila *et al.* 2013) was used in this work. Seeds were retrieved from USDA-ARS-NCRPIS (Iowa State University, Regional Plant Introduction Station, Ames, Iowa, United States, 50011-1170). The panel was previously evaluated for response to *F. verticillioides* inoculation (FIS) by Stagnati and co-workers (2019). In that work, data about seedling germination were exclusively used in order to remove lines with <50% germination percentage (Stagnati *et al.*, 2019). Accordingly, only 230 out of 302 inbred lines of the population having sufficient germination rate were screened and considered for further analysis in the present study (Table S1).

To artificially infect mature kernels and evaluate the effect of *F. verticillioides* on some vegetative traits of maize seedlings, the Rolled Towel Assay (RTA) phenotyping method was used (Ellis *et al.* 2011; Lanubile *et al.* 2015; Stagnati *et al.* 2019). For each inbred line, seeds with similar size and shape, and without visible damage, were selected. To reduce as much as possible the presence of contaminating fungi, seeds were surface-sterilized as previously described (Stagnati *et al.* 2019). RTA were prepared using 10 seeds for each inbred per towel of moistened geminating paper (Anchor Paper, Saint Paul, MN). Kernels were inoculated on the embryo side near the pedicel with 100 μl of 1x10^6^ conidial suspension of *F. verticillioides* ITEM10027 (MPVP 294) and sterilized distilled water for treated and control towels, respectively. Incubation was performed for 7 days in the dark at 25° as previously reported (Bernardi *et al.* 2018; Stagnati *et al.* 2019).

For each seedling, plant length (PL) from the tip to the end of the longest root, and fresh plant weight (PW) of maize genotypes in control (C) and inoculated (I) conditions were calculated. Germination percentage was evaluated as reported by Stagnati *et al.* (2019).

### Association analysis, candidate gene discovery and linkage disequilibrium analysis

The ZeaGBSv2.7 set of SNP derived from Genotyping By Sequencing and available at Panzea (www.panzea.org) was used. Monomorphic and multiallelic SNPs and INDELs were discarded. Imputation was performed using the software Beagle 4.1 (Browning and Browning 2016). Heterozygous were set as missing data and removed the SNPs if >20% of missing data and minor allele frequency (MAF) <5%. After the final imputation, a set of 226,446 SNPs was used for association analysis.

Marker pairs with genotypic correlation higher than *r* = 0.5 were pruned according to a linkage disequilibrium based approach using the software Plink v1.07 (Purcell *et al.* 2007). A subset of around 100kb SNPs was obtained to compute the additive relationship matrix (K matrix) for the 230 inbred using TASSEL v5.2.25 (Bradbury *et al.* 2007).

Genome Wide Association Analysis (GWAS) was performed in Tassel (version 5.2.25). The mixed linear model (MLM) fitted by Tassel was:

y=Xβ+Zu+e

Where y is the vector of phenotypes, β is a vector of the overall mean and the fixed effect estimate of an individual SNP, *u* is a vector of random line additive genetic effects, X and Z represent incidence matrices, and *e* is a vector of random residuals. Variance of random line effects was modeled as Var (u) = $K\sigma_{a}^{2}$, where $\sigma_{a}^{2}$ is the estimated additive polygenic variance. The optimum compression level option (compressed MLM) was used (Zhang *et al.* 2010).

To identify robust SNP associations, a resampling procedure was performed. In each of 100 data resamples, a random sample of 80% of inbred lines was selected from the population, GWAS was performed on the subset of lines. Only SNP markers determined as significant at *P* < 1x10^−4^ within at least 30% of data subsamples, *i.e.*, a resample model inclusion probability (RMIP) threshold of 0.30, were considered as significant (Samayoa *et al.* 2015; Stagnati *et al.* 2019). Data manipulations and visualizations were performed using R software (R Core Team 2016).

Genes containing or close to associated SNPs were detected using Maize GDB genome browser using the B73 RefGen_V3 as the reference genome to place SNPs and genes avoiding low confidence genes and transposable elements. The function and conserved domains of proteins encoded by genes identified were retrieved submitting sequences to NCBI-protein BLAST (https://blast.ncbi.nlm.nih.gov) and restricting the search to *Zea mays* (taxid:4577). For SNPs positioned inside genes the possible effect on protein sequence was inquired. Introns and translated regions were identified using *GeneWise* online tool (http://www.ebi.ac.uk), coding sequence were translated using ExPASy (http://web.expasy.org/translate) and amino-acid variations were detected aligning the reference protein retrieved from MaizeGBD, and the SNP derived protein using MultAlin (http://multalin.toulouse.inra.fr).

Linkage disequilibrium (LD) measures (*r*^2^) were estimated using TASSEL version 5.0 between each significant SNP and other SNPs within a surrounding window of 60 adjacent SNPs (Samayoa *et al.* 2015; Stagnati *et al.* 2019). For clusters of tightly linked significant SNPs, LD analysis was computed for one of them; positions were considered linked to significant SNPs if *r*^2^ > 0.5 and the distance between the position and the SNP resulting from GWAS was greater than 2 Kb.

### Data availability

All supporting data are included as supplemental files and are available at Figshare: https://figshare.com/s/54bb47a6abf418c859b9. Table S1. (Excel file) List of germplasm and phenotypic values. Table S2. (DOCX file) Table of LD analysis.

## Results and Discussion

### Phenotypic data

**Figure 1:**
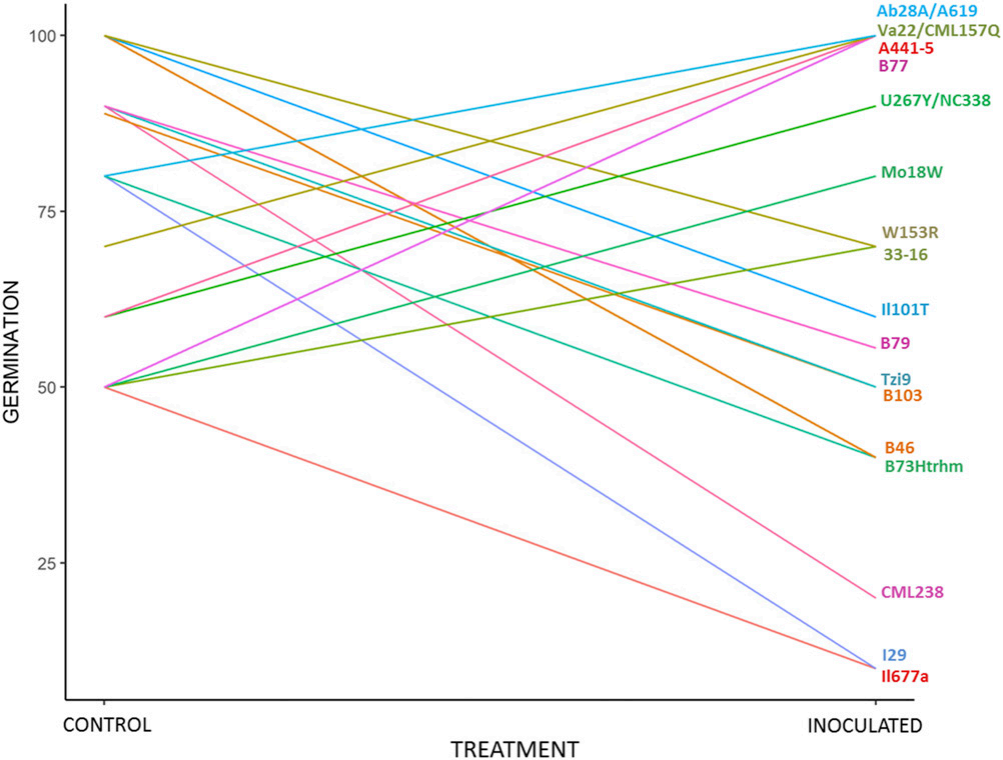
Effect of inoculation with *Fusarium verticillioides* on germination percentage compared to control, in kernels of selected maize lines.

Seed germination was affected in different ways by artificial inoculation (Figure 1; Table S1). In 59 cases, germination rate were higher in the inoculated towels, whereas in 87 lines the germination was negatively affected by the fungus. Similar numbers were also observed in 84 lines without and with infection. Figure 1 illustrates the change in germination due to inoculation for 20 inbreds with the most extreme reactions after inoculation. The germination of inbreds I29 and CML238 was reduced by 70% resulting in 10 and 20%, respectively, after inoculation; germination of Il677a was reduced from 50 to 10% by the inoculation. I29 (a popcorn) and Il677a (a sweet corn) were the inbreds with the lowest germination rate after inoculation and both showed increased disease severity values following inoculation (Stagnati *et al.* 2019).

The ability of *F. verticillioides* to influence seed germination and vigor in maize was previously explored. Some studies reported that the fungus can condition host fitness promoting growth and inducing defense mechanisms such as lignin deposition in the cell wall (Yates *et al.* 1997). Seedlings grown from seeds inoculated with *F. verticillioides* showed reduced growth at early stages of infection, but by 21 days after planting the inoculated plants adjusted or overtook the mock plants in shoot diameter, root growth and dry weight (Yates *et al.* 1997). Additionally, previous reports in literature highlighted how maize seed lots with high incidence of this fungus (inoculum potential) underwent little or no reduction in germination or seedling growth, while others were seriously compromised by the fungus (Munkvold *et al.* 1997; Oren *et al.* 2003; Machado *et al.* 2013). Beside the fungal inoculum potential in a seed lot, further sources of variation in seedling germination should be taken in account, such as the genetic nature of the several inbred employed in this study that can have influenced the establishment of the maize-fungal association, determining even a beneficial effect to some hosting lines.

**Figure 2:**
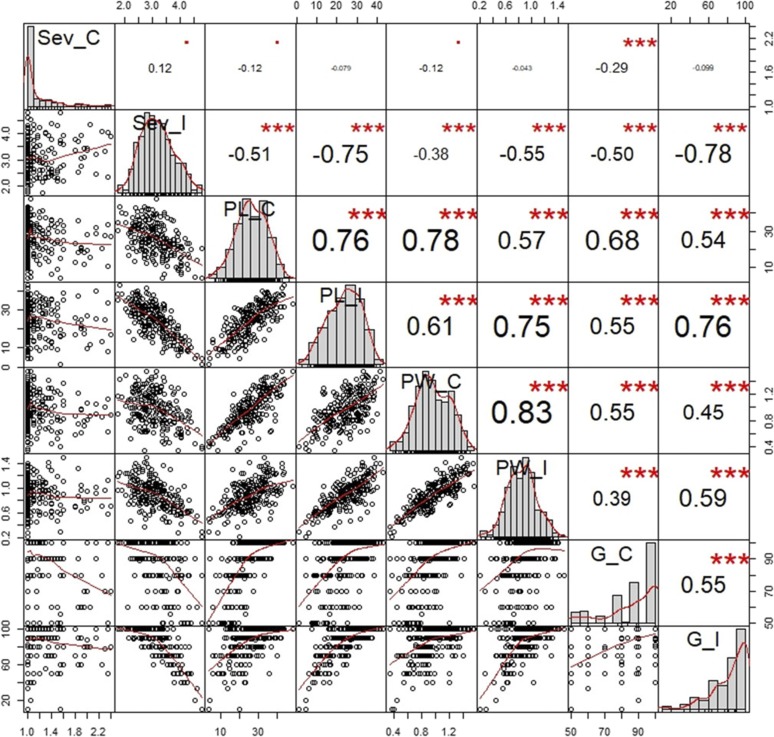
Correlation among disease severity (SEV; Stagnati *et al.* 2019), plant length (PL), plant weight (PW), germination percentage (G) traits measured in the control (C) or inoculated (I) plants. The distribution of each trait is shown on the diagonal. On the bottom of the diagonal the bivariate scatter plots with a fitted line are displayed. On the top of the diagonal the value of the correlation plus the significance level are reported as stars (*** p-value 0.001).

No significant correlations were observed in non-inoculated assays between either PL_C or PW_C and the FIS severity (Sev_C), previously evaluated in Stagnati *et al.* (2019), whereas a moderately negative correlation was found for the germination percentage (G_C; Figure 2). As expected, after *F. verticillioides* infection, PL_I, PW_I and G_I showed significant negative correlations with Sev_I (*r* =-0.75, -0.55 and -0.78 for PL_I, PW_I and G_I, respectively; Figure 2). In line with these achievements, previous studies reported that *F. verticillioides* infection diminished germination of maize kernels and hampered seedling growth and vigor (Van Wyk *et al.* 1988; Septiani *et al.* 2019).

Furthermore, seedling traits (length, weight and germination) of both inoculated and control assays were significantly correlated each other (Figure 2). This finding could be explained by the inherent inbred line characteristics like seed size, weight and seedling growth vigor.

### Association analysis and SNP discovery

**Figure 3:**
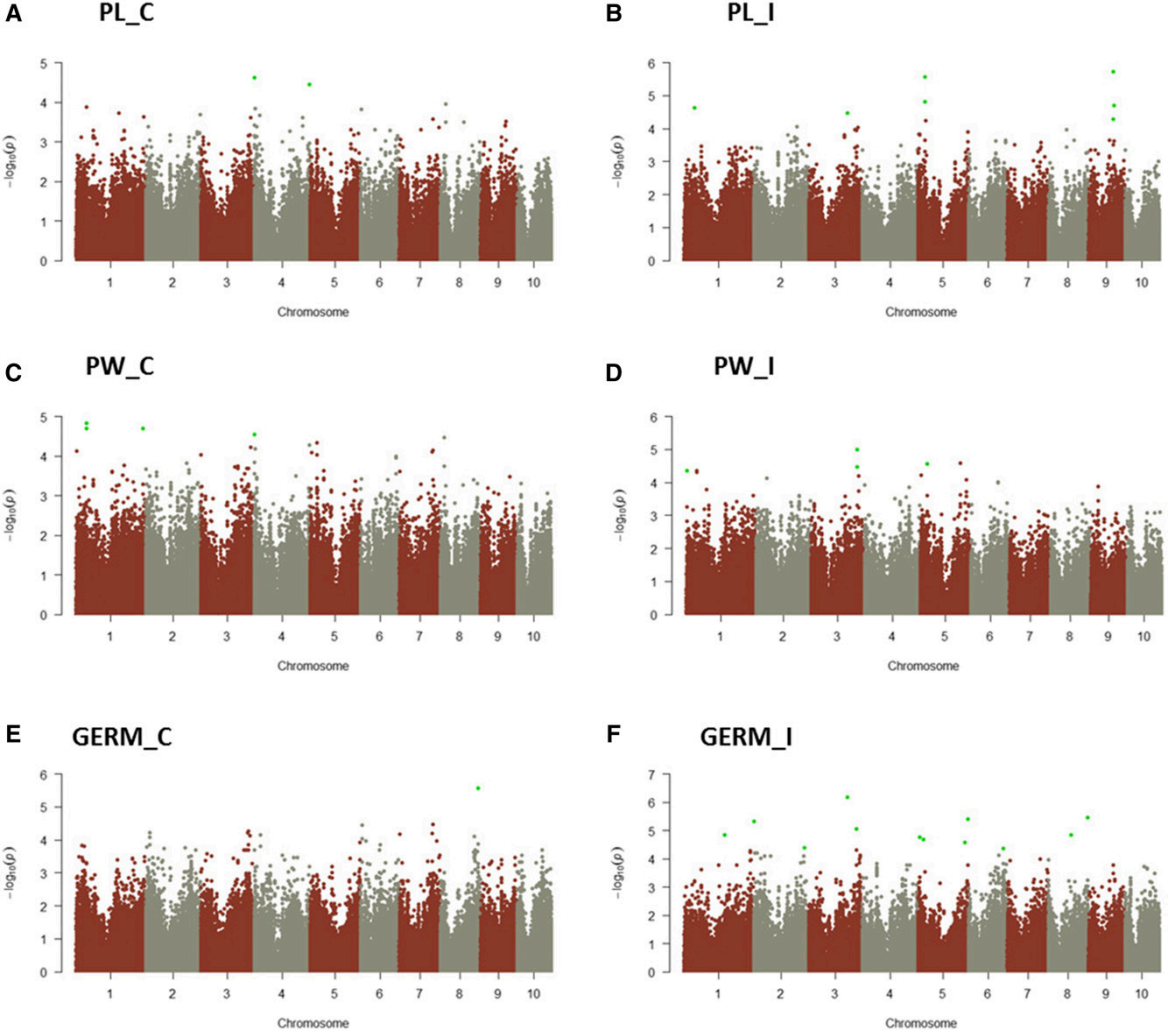
Manhattan plots of the traits PL_C (A), PL_I (B), PW_C (C), PW_I (D), GERM_C (E) and GERM_I (F). SNPs significantly associated to the traits are indicated by green dots.

GWAS analysis resulted in 42 SNPs, distributed across all chromosomes, significantly associated with the traits examined. 35 SNPs were located in 21 genes, while the remaining 7 were intergenic, but within 1.1 kb of known genes (Tables 1 and 2; Figure 3). A total of 25 genes were identified as containing or close to SNPs significantly associated with at least one of the measured traits. Six SNPs were in common between two traits each, *i.e.*, four SNPs for PL_I and PW_C; one SNP for PL_I and GERM_I and one SNP for PL_C and PW_C.

**Table 1:** Chromosome (CHR) location, number of genes and SNPs localized inside (IN) or outside (OUT) predicted coding regions of genes significantly associated with the traits PL_C, PL_I, PW_C, PW_I, GERM_C and GERM_I

TRAIT	CHR	GENES	SNPs	IN	OUT
**PL_C**	4	2	2	2	0
**PL_I**	1	1	4	3	1
**PL_I**	3	1	1	1	0
**PL_I**	5	1	3	3	0
**PL_I**	9	3	3	2	1
**PW_C**	1	2	5	3	2
**PW_C**	4	1	1	1	0
**PW_I**	1	1	1	1	0
**PW_I**	3	1	7	7	0
**PW_I**	5	1	1	1	0
**GERM_C**	8	1	1	1	0
**GERM_I**	1	1	1	1	0
**GERM_I**	2	2	2	1	1
**GERM_I**	3	3	3	2	1
**GERM_I**	5	4	4	4	0
**GERM_I**	6	1	1	1	0
**GERM_I**	8	2	2	1	1

**Table 2:** Marker name, allelic variants (SNP), number of lines carrying each allelic variant (OBS), additive effect, *p*-values for the association between the SNP and the phenotype, resample model inclusion probability (RMIP) and proportion of the phenotypic variance associated with the SNP (R^2^) referred to the traits PL_C, PL_I, PW_C, PW_I, GERM_C and GERM_I

N°	Trait	Marker^a^	SNP^b^	OBS^c^	Additive effect^d^	P-value	R^2^	RMIP
1	PL_C	S4_237392707	A/G	216/14	−5.50	3.85*10^−06^	0.08	0.4
2 – 19	PL_C	S4_768454	G/A	150/76	−2.89	2.52*10^−06^	0.09	0.3
3 – 15	PL_I	S1_45515811	A/G	105/23	−2.63	5.19*10^−07^	0.08	0.4
4 – 16	PL_I	S1_45515933	G/T	122/106	2.64	5.86*10^−07^	0.08	0.4
5 – 17	PL_I	S1_45515951	T/C	122/106	2.64	5.86*10^−07^	0.08	0.4
6 – 18	PL_I	S1_45516086	G/C	106/122	2.64	5.86*10^−07^	0.08	0.4
7 – 33	PL_I	S3_166796369	G/A	217/12	−5.65	5.56*10^−06^	0.07	0.4
8	PL_I	S5_30755308	G/T	155/74	−2.84	1.01*10^−06^	0.08	0.5
9	PL_I	S5_30755309	C/A	154/75	−3.08	1.31*10^−07^	0.10	0.8
10	PL_I	S5_30755313	G/T	154/75	−3.08	1.31*10^−07^	0.10	0.8
11	PL_I	S9_105426127	G/A	192/37	−4.43	1.60*10^−07^	0.10	0.9
12	PL_I	S9_105564038	C/A	183/47	−3.20	2.00*10^−06^	0.07	0.4
13	PL_I	S9_108462691	C/T	209/20	−4.76	3.79*10^−07^	0.08	0.4
14	PW_C	S1_290520672	G/T	188/38	−0.09	2.18*10^−06^	0.08	0.5
15 – 3	PW_C	S1_45515811	G/A	123/105	0.07	2.29*10^−07^	0.08	0.6
16 – 4	PW_C	S1_45515933	G/T	122/106	0.07	3.30*10^−07^	0.08	0.6
17 – 5	PW_C	S1_45515951	T/C	106/122	0.07	3.30*10^−07^	0.08	0.6
18 – 6	PW_C	S1_45516086	G/C	106/122	0.07	3.30*10^−07^	0.08	0.6
19 - 2	PW_C	S4_768454	G/A	150/76	−0.07	2.72*10^−06^	0.08	0.5
20	PW_I	S1_3366706	T/C	198/30	0.10	1.34*10^−06^	0.07	0.4
21	PW_I	S3_200433261	C/T	162/65	−0.07	3.39*10^−06^	0.08	0.3
22	PW_I	S3_200433799	C/T	152/77	−0.08	1.78*10^−06^	0.08	0.5
23	PW_I	S3_200433801	G/C	152/77	−0.08	1.78*10^−06^	0.08	0.5
24	PW_I	S3_200433802	A/C	152/77	−0.08	1.78*10^−06^	0.08	0.5
25	PW_I	S3_200433803	T/C	152/77	−0.08	1.78*10^−06^	0.08	0.5
26	PW_I	S3_200433807	T/C	152/77	−0.08	1.78*10^−06^	0.08	0.5
27	PW_I	S3_200433834	G/A	152/77	−0.08	1.78*10^−06^	0.08	0.5
28	PW_I	S5_31445260	T/C	131/98	0.07	7.31*10^−06^	0.08	0.4
29	GERM_C	S8_164380034	G/T	158/68	−5.54	2.53*10^−08^	0.10	0.7
30	GERM_I	S1_174846151	C/T	214/16	10.77	6.61*10^−08^	0.08	0.5
31	GERM_I	S2_2069896	G/A	215/14	−12.08	2.71*10^−08^	0.09	0.6
32	GERM_I	S2_219731457	A/G	182/44	−6.82	6.16*10^−06^	0.07	0.4
33-7	GERM_I	S3_166796369	G/A	217/12	−14.21	5.72*10^−09^	0.11	0.8
34	GERM_I	S3_206944106	A/G	217/13	12.03	1.53*10^−07^	0.09	0.5
35	GERM_I	S3_206950032	G/T	217/13	12.03	1.53*10^−07^	0.09	0.5
36	GERM_I	S5_201596318	C/G	212/16	−10.14	9.04*10^−07^	0.08	0.5
37	GERM_I	S5_216440094	C/T	216/13	−12.78	9.66*10^−08^	0.09	0.7
38	GERM_I	S5_24609625	G/C	119/108	−5.63	9.80*10^−07^	0.08	0.3
39	GERM_I	S5_6906047	T/C	179/46	−7.19	4.97*10^−06^	0.08	0.3
40	GERM_I	S6_150075477	G/A	163/66	−6.07	4.87*10^−06^	0.07	0.4
41	GERM_I	S8_100397575	T/C	214/16	−10.40	4.88*10^−07^	0.08	0.5
42	GERM_I	S8_171745673	A/G	203/24	−11.29	2.52*10^−07^	0.09	0.6

For the trait PL_C only two SNP associations were discovered, both on chromosome 4, but inside two different genes (Figure 3A). Eleven SNPs were found associated with the trait PL_I (Figure 3B): including a cluster of four SNPs in or near the same gene on chromosome 1, one SNP on chromosome 3, three SNPs inside the same gene on chromosome 5 and further three SNPs on chromosome 9. Five SNPs in or near two different genes on chromosome 1 and a SNP on chromosome 4 were associated with PW_C (Figure 3C). For PW_I an associated SNP was found in a gene on chromosome 1, seven SNPs were in a gene on chromosome 3 and one SNP was in a gene on chromosome 5 (Figure 3D).

For the trait GERM_C only one significant SNP was identified and it was inside a gene on chromosome 8 (Figure 3E). For GERM_I thirteen SNPs were observed (Figure 3F). One is located in a gene on chromosome 1, two SNPs highlighted two genes on chromosome 2 and three SNPs were found on chromosome 3 associated with 3 genes. On chromosome 4, 4 SNPs were identified inside the same number of genes; on chromosome 6 a SNP highlighted one gene and on chromosome 8 two SNPs, one inside and one close by two different genes.

The highest number of genes was identified on chromosome 5, while chromosome 6 had only one gene associated to disease traits. Genes highlighted were generally at the end of the chromosome than in centromere or pericentromeric regions. No SNPs on chromosomes 7 and 10 were associated to traits.

### Genes associated with PL, PW and GERM traits

Table 3 reports all genes containing or close to SNP markers deriving from GWAS and significantly associated to the traits length, weight and germination in control and inoculated seedlings. A SNP (S4_768454) was found inside the gene GRMZM2G058394 and associated with traits PL_C and PW_C. This gene encodes a putative thaumatin like-protein (TLP). TLPs are implicated in host defense and in various developmental processes. These genes are expressed in response to environmental perturbations, drought, cold and pathogen attacks. Therefore, TLPs are classified as pathogenesis related protein (PR) (Marchler-Bauer *et al.* 2015). TLPs are universal in plants. The antifungal activity of TLPs is well reported (Liu *et al.* 2010; Sharma *et al.* 2020) and for some of them the binding activity to β-1,3-D-glucanase is described. β-1,3-D-glucan is a key component of fungal cell wall and the activity of this enzyme is important in the breakdown of fungal cell (Liu *et al.* 2010; Sharma *et al.* 2020). The TLP family also includes a xylanase inhibitor (Liu *et al.* 2010; Fierens *et al.* 2007); considering that xylan is an important component of plant cell wall, the xylanase activity of TLPs is one of the mechanisms by which these proteins are involved in plant-defense mechanism (Liu *et al.* 2010). In maize ears and silks infected by *F. verticillioides*, thaumatin gene had lower expression ratios in resistant lines compared to susceptible ones. In contrast, kernels of the resistant lines showed higher gene expression before fungal infection, highlighting the key role of constitutive resistance in limiting pathogen attack (Lanubile *et al.* 2010, 2012a,b; Maschietto *et al.* 2016). The SNP found in this gene did not cause an amino-acid change in the protein.

**Table 3:** Genes containing or near SNPs significantly associated with the traits PL, PW and GERM in control and *Fusarium* inoculated seedlings. For each marker, the genomic position (pos_AGP3), the surrounding or adjacent gene, the type and the conserved domain of the encoded protein (where available) and the amino-acid variation caused by the SNP are reported

N°	Gene_id	Strand^1^	pos_AGP3	Description	Conserved domains	Genic^2^	Amino-acid^3^
1	GRMZM5G851807	+	237936341	Putative DNA-directed RNA polymerase family protein	PRK13042, superantigen-like protein, DUF3223, SPT5 elongation factor	TRUE	E_UTR
2–19	GRMZM2G058394	+	768454	Putative thaumatin domain family protein	Glycoside hydrolase family 64	TRUE	No change
3–15	GRMZM2G087186	—	45520333	Pyruvate decarboxylase isozyme 3	Pyruvate decarboxylase domains	TRUE	No change
4–16			45520455			TRUE	E_UTR
5–17			45520473			TRUE	E_UTR
6–18			45520608			107	NA
7–33	GRMZM2G141638	+	166839392	Putative AP2/EREBP transcription factor superfamily protein	AP2 domain	TRUE	No change (T1), E_UTR(T2)
8	GRMZM2G027232	+	30775149	50S ribosomal protein L11;	Ribosomal protein	TRUE	E_UTR
9			30775150			TRUE	E_UTR
10			30775154			TRUE	E_UTR
11	GRMZM2G031370	+	106433186	Uncharacterized protein	RABGAP_TBC domain GTPase activator protein in yeast	TRUE	E_UTR
12	GRMZM2G058162	—	106571097	putative ubiquitin-conjugating enzyme family	UBCc superfamily	400	NA
13	GRMZM2G338376	—	108482377	Uncharacterized protein	Serine threonine protein kinase	TRUE	Intron
14	GRMZM2G012224	+	290593516	Putative uncharacterized protein	DUF642/ and CBM_4_9_Carbohydrate binding domain	1114	NA
20	GRMZM2G426802	—	3359556	Signal transducer	WD40 signal transducer,	TRUE	Intron
21	GRMZM5G800586	—	200505296	Uncharacterized protein	BURP domain of unknown function	TRUE	Intron
22			200505834			TRUE	E_UTR
23			200505836			TRUE	E_UTR
24			200505837			TRUE	E_UTR
25			200505838			TRUE	E_UTR
26			200505842			TRUE	E_UTR
27			200505869			TRUE	E_UTR
28	GRMZM2G154954	+	31465101	Glycine rich protein 3	No conserved domains	TRUE	No change
29	GRMZM2G024799	—	163929482	SF16 protein	No conserved domains	TRUE	E_UTR
30	GRMZM2G008250	+	174872171	Uncharacterized protein	No conserved domains	TRUE	No change
31	GRMZM2G040627	+	2072269	Uncharacterized protein	Uracil-DNA glycosylase /DNA repair	TRUE	No change
32	GRMZM2G025783	—	220400327	Protein kinase Kelch repeat:Kelch	Kelch and Fbox associated with LRR	484	NA
34	GRMZM2G497710	+	207023192	Uncharacterized protein	Wall-associated receptor kinase galacturonan-binding, GUB_WAK_bind domain.	TRUE	E_UTR
35	GRMZM2G028568	+	207029118	Uncharacterized protein	Serine threonine protein kinase(stress response/ GUB_WAK domain	45	NA
36	GRMZM2G071970	+	6914037	Brittle stalk-2-like protein 4	COBRA-like protein	TRUE	E_UTR
37	GRMZM2G032648	—	24629650	Uncharacterized protein	CBS domain	TRUE	No change (T1) E_UTR (T2)
38	GRMZM2G125432	+	201652485	NA	Putative SET-domain protein	TRUE	E_UTR (T1-2)
39	GRMZM2G171801	—	216492681	NA	DEAD-box ATP-dependent RNA helicase 48-like	TRUE	R317K
40	GRMZM2G129288	+	150269434	Uncharacterized protein	TUB and F-box domain	TRUE	Intron (T1-2-3), E_UTR (T4-5)
41	GRMZM2G126010	—	99908826	Actin-1	Nucleotide-Binding Domain of the sugar kinase/HSP70/actin superfamily	TRUE	E_UTR
42	GRMZM2G139460	+	171298650	Uncharacterized protein	DUF4228	229	NA

1: indicates if the gene is on the forward (+) or reverse (-) strand. 2: indicates if the marker is inside (TRUE) a gene, otherwise the distance from the closest gene is reported. 3: indicate the amino acid variation (reference amino acid/position of variation/new amino acid), SNPs located in un-translated regions are reported (E_UTR); if the SNP had different consequences on different transcripts, of the same gene, that of interest is reported, NA means that the marker is not inside the gene.

A SNP in the gene GRMZM2G338376, which encodes a protein kinase with domains common to the receptor like kinase (RLK), was associated with PL_I. RLKs are surface transmembrane receptors able to perceive different signals and the presence of pathogens. The RLK family includes the BAK1 that is involved in brassinosteroid-mediated plant development. Interestingly, in previous studies this gene was highly expressed in maize coleoptile at V1 stage (Sekhon *et al.* 2011) and presented a strong modulation in resistant ears at 72 hr after *F. verticillioides* infection (Lanubile *et al.* 2014a; Wang *et al.* 2016). Furthermore, previous association mapping analysis identified BAK1 in two QTL for *Fusarium* seedling rot resistance (qFSR4.2) and seedling weight (qSWT3.1) in inoculated kernels of a Multi-parent Advance Generation Intercross (MAGIC) maize population (Septiani *et al.* 2019).

GRMZM2G154954, including the SNP S5_31445260 associated with PW_I, encodes the glycine rich protein 3. These proteins are key component of plant cell wall, and their expression was described in response to lesions caused by biotic and abiotic factors, in defense against *Pseudomonas syringae*, and two glycine rich peptides isolates from *Capsella bursa-pastoris* were active against several bacteria and fungi (Mangeon *et al.* 2010).

The SNP S2_219731457 associated with GERM_I was near the gene GRMZM2G025783. The protein encoded by this gene contains the F-box domain that mediates protein-protein interactions in several contests and in polyubiquitination. The F-box associated to the Tub superfamily domain was found also in the gene GRMZM2G129288, which was highlighted by the SNP 40, located in the UTR of all the 5′ transcripts for this gene (Marchler-Bauer *et al.* 2015).

Additional SNPs associated with GERM_I were identified in the GRMZM2G028568 and GRMZM2G125432 genes. GRMZM2G028568 encodes an uncharacterized protein with the Serine/threonine kinase domain, involved in plant resistance, and the wall-associated receptor kinase galacturonan-binding, which is an extracellular part of the serine/threonine kinase binding cell-wall pectins. This domain is responsible of the phosphorylation of Serine-Threonine residues and implicated in plant resistance (Afzal *et al.* 2008). GRMZM2G125432 encodes a protein with Pre-SET and SET domains. Pre-SET domain stabilizes SET domain, which is a lysine methyltransferase involved in protein-protein interactions (Thorstensen *et al.* 2011). A group of SET domains have been classified as PR proteins.

### Linkage disequilibrium analysis

Linkage disequilibrium **(**LD) analysis was performed in a window of 60 adjacent SNPs on the same chromosome to identify correlations between markers. Genome-wide, 4 SNPs or groups of SNPs were detected in LD of *r*^2^ greater than or equal to 0.5 to significantly associated markers (Table S2). Generally, for each trait-associated SNP examined, from 1 to 3 SNPs were detected to be in LD *r*^2^ > 0.5.

Regarding the trait PW_I, only the gene GRMZM5G800586 was found both by GWAS and LD analysis; this gene encodes a protein of unknown function.

For the trait GERM_I, three positions were found in LD in the gene GRMZM2G497710 that was also identified by GWAS; also GRMZM2G125432 already reported as containing a SNP was the closest to a position found by LD analysis.

Genome-wide, three genes were identified by both analyses (GWAS and LD); while LD analysis exclusively revealed only one new gene.

### Comparison between genes detected by GWAS and QTL for resistance to Fusarium

**Figure 4:**
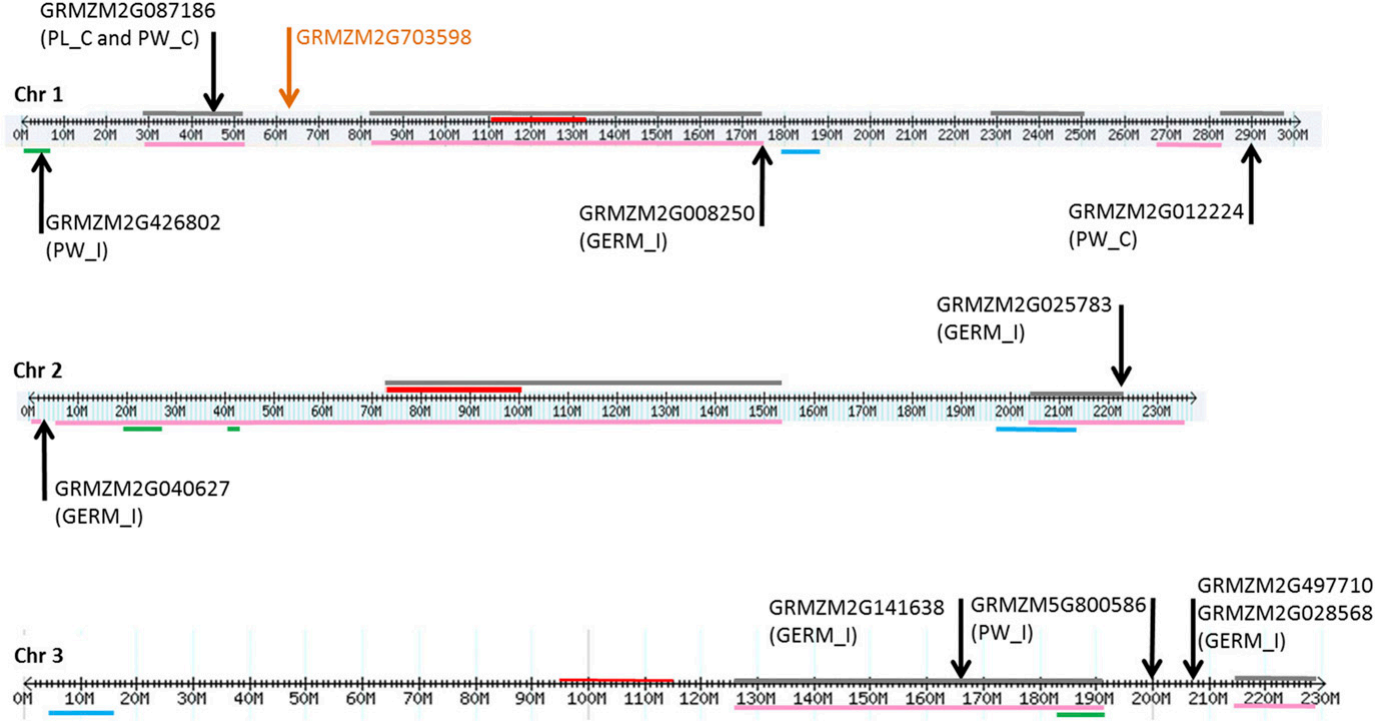
Localization of QTL and genes containing or adjacent to SNPs identified by GWAS on chromosomes 1, 2 and 3. Genes associated with different traits (PL_C; PL_I; PW_C; PW_I; GERM_C; GERM_I) from this study are indicated by the black arrow. Candidate genes from Zila *et al*. (2013) are indicated by the orange arrow. Horizontal lines of different colors represent different QTL intervals for resistance to *Fusarium* and fumonisin accumulation identified by previous reports: pink and gray (*Fusarium* ear rot and fumonisins from Lanubile *et al.* 2014b and Maschietto *et al.* 2017, respectively); green (*Fusarium* seed rot from Ju *et al.* 2017); blue (*Fusarium* seedling rot, plant length and weight from Septiani *et al.* 2019). The centromere is indicated by a red label on the chromosome.

**Figure 5:**
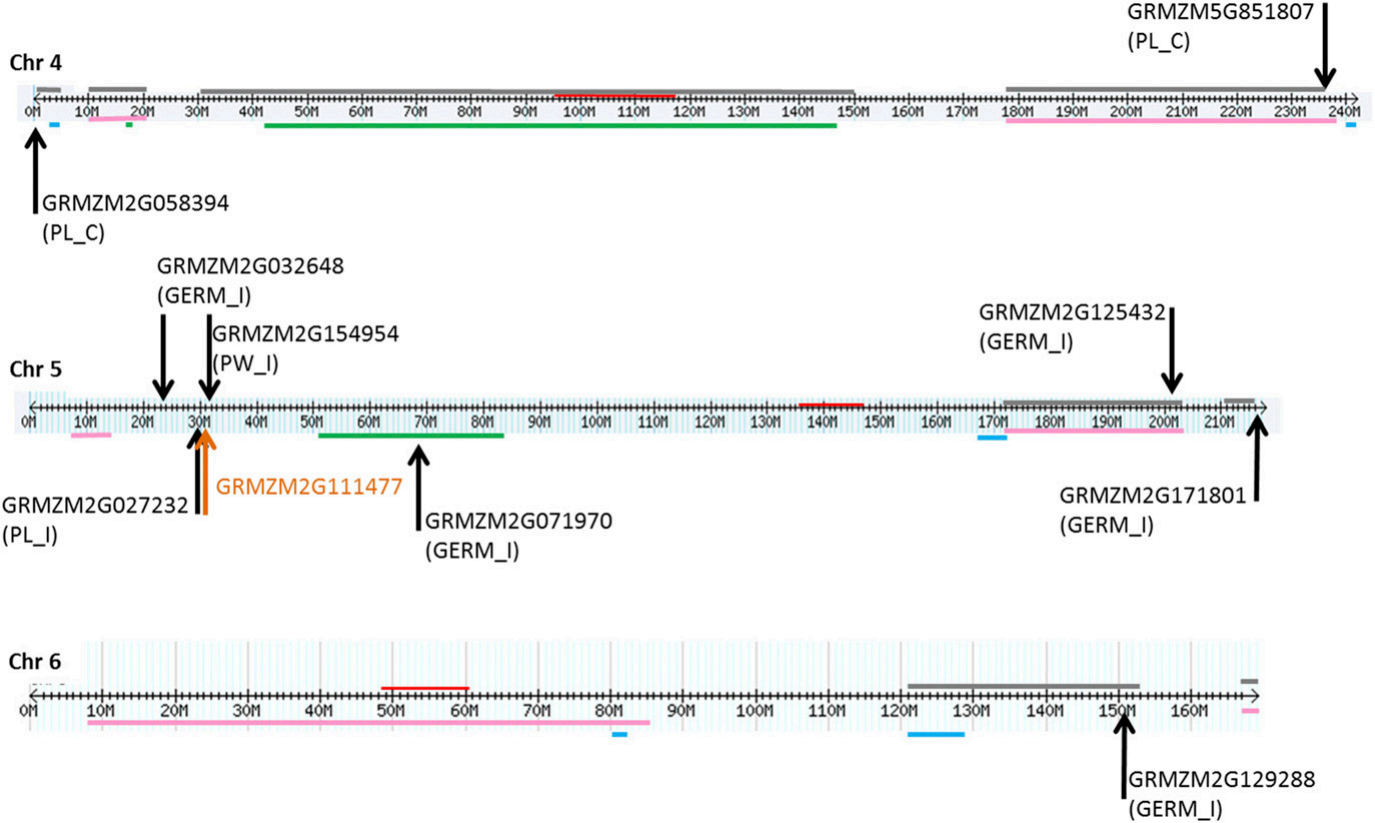
Localization of QTL and genes containing or adjacent to SNPs identified by GWAS on chromosomes 4, 5 and 6. Genes associated with different traits (PL_C; PL_I; PW_C; PW_I; GERM_C; GERM_I) from this study are indicated by the black arrow. Candidate genes from Zila *et al.* (2013) are indicated by the orange arrow. Horizontal lines of different colors represent different QTL intervals for resistance to *Fusarium* and fumonisin accumulation identified by previous reports: pink and gray (*Fusarium* ear rot and fumonisins from Lanubile *et al.* 2014b and Maschietto *et al.* 2017, respectively); green (*Fusarium* seed rot from Ju *et al.* 2017); blue (*Fusarium* seedling rot, plant length and weight from Septiani *et al.* 2019). The centromere is indicated by a red label on the chromosome.

**Figure 6:**
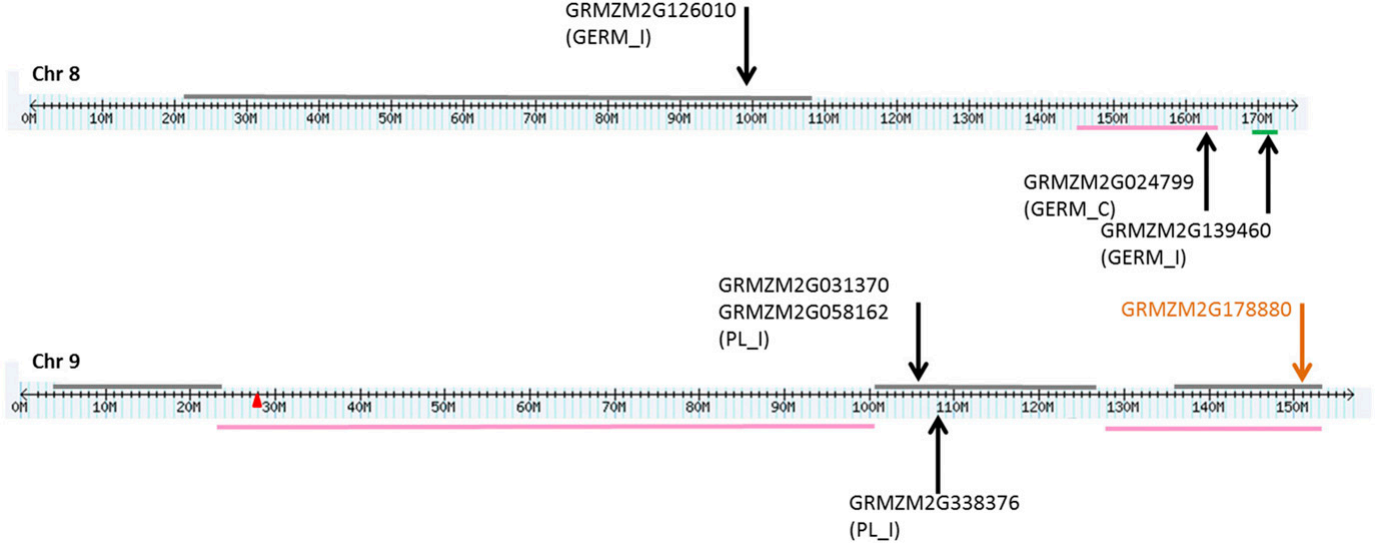
Localization of QTL and genes containing or adjacent to SNPs identified by GWAS on chromosomes 8 and 9. Genes associated with different traits (PL_C; PL_I; PW_C; PW_I; GERM_C; GERM_I) from this study are indicated by the black arrow. Candidate genes from Zila *et al.* (2013) are indicated by the orange arrow. Horizontal lines of different colors represent different QTL intervals for resistance to *Fusarium* and fumonisin accumulation identified by previous reports: pink and gray (*Fusarium* ear rot and fumonisins from Lanubile *et al.* 2014b and Maschietto *et al.* 2017, respectively); green (*Fusarium* seed rot from Ju *et al.* 2017); blue (*Fusarium* seedling rot, plant length and weight from Septiani *et al.* 2019). The centromere is indicated by a red label on the chromosome.

Since QTL often refer to chromosomal regions that may span several Mb of the genome, in which several genes may be located, genes overlapping previously reported QTL involved in resistance to FER, fumonisin accumulation, *Fusarium* seed rot (FSR) and seedling traits, including seedling length, weight and FIS, were investigated (Figures 4-6). Genes containing or adjacent to significant SNPs identified by GWAS analysis were located on chromosomes according to their physical position. In parallel, known QTL for the traits mentioned above were located on chromosomes according to ‘bin’ positions (Zila *et al.* 2013; Lanubile *et al.* 2014b; Ju *et al.* 2017; Maschietto *et al.* 2017; Septiani *et al.* 2019).

On chromosome 1, genes were well distributed and associated for all traits (Figure 4). The genes GRMZM2G012224 and GRMZM2G087186 associated with PW_C, the later one also associated with PL_C, and GRMZM2G008250 with GERM_I were located inside known QTL found for both FER and fumonisin accumulation resistance. Additionally, the gene GRMZM2G426802 related to PW_I overlapped the QTL for FSR. In a previous work, Zila *et al.* (2013) reported the gene GRMZM2G703598 associated with FER resistance on chromosome 1 detected in the same association population employed in this study and assayed in field conditions.This gene did not co-localize within any QTL in the map (Figure 4).

Two genes (GRMZM2G040627 and GRMZM2G025783) on chromosome 2 and one (GRMZM2G141638) on chromosome 3 related to GERM_I overlapped two QTL regions reported for FER and fumonisin accumulation (Figure 4).

The genes associated with PL_C, GRMZM2G058394 and GRMZM5G851807, were located at the ends of QTL reported for FER and fumonisin resistance on chromosome 4 (Figure 5).

On chromosome 5, two genes (GRMZM2G125432 and GRMZM2G171801) related to GERM_I were located inside QTL reported for FER and fumonisin resistance. One more gene (GRMZM2G071970) associated with the same trait was located inside a known QTL involved in FSR resistance (Figure 5). Moreover, the gene GRMZM2G11147 identified by Zila *et al.* (2013) was very close to the genes GRMZM2G027232 and GRMZM2G154954 for PL_I and PW_I, respectively.

One gene related to GERM_I (GRMZM2G129288) was located inside a QTL reported for fumonisin accumulation on chromosome 6 (Figure 5).

Two genes associated with GERM_I traits (GRMZM2G126010 and GRMZM2G139460) overlapped with QTL intervals for resistance to fumonisin accumulation and FSR on chromosome 8, respectively, and one additional gene for GERM_C (GRMZM2G024799) was in the QTL for FER (Figure 6).

On chromosome 9, three genes (GRMZM2G031370, GRMZM2G058162 and GRMZM2G338376) were found associated with PL_I inside a QTL reported for fumonisin accumulation (Figure 6).

No gene associations were observed on chromosomes 7 and 10.

In the recent work by Ju *et al.* (2017), three QTL for FSR were found associated to three genes identified in this study, two for GERM_I and one for PW_I (Figures 4-6). This finding once again confirms how *F. verticillioides* infection limits germination of maize kernels and reduces seedling weight and vigor. Conversely, no correspondence was observed with QTL previously described by Septiani *et al.* (2019), where the seedling weight and length as well as *Fusarium* seedling rot traits were evaluated by the same phenotyping method. This could be explained by the different genetic materials employed for mapping in the two researches (association mapping panel *vs.* multi-parent population-MAGIC) and the use of diverse genome assemblies.

## Conclusions

In this work, a bioassay was applied to the maize association panel tested for seedling related traits to *F. verticillioides* resistance. GWAS analysis revealed 42 SNPs associated with three phenotypic traits: plant seedling length (PL), plant seedling weight (PW) and germination rate. In total, 25 genes were found associated with the SNPs and many of these genes had a role in disease response.

Comparison between genes found in our GWAS analysis and previous QTL mapping studies reported for resistance to FER, fumonisin accumulation and seedling traits revealed that the majority of the genes, associated with the SNPs, localized inside QTL for these traits.

These findings will help us to better understand the existing complex interaction between maize and *F. verticillioides* in order to improve genomic selection for *Fusarium* resistance at the seedling stage. Functional validation of the most recurring candidate genes will be required to verify their role in the pathways to hamper fungal infection in maize.
